# Photoluminescence of compact GeSi quantum dot groups with increased probability of finding an electron in Ge

**DOI:** 10.1038/s41598-020-64098-x

**Published:** 2020-06-09

**Authors:** A. F. Zinovieva, V. A. Zinovyev, A. V. Nenashev, S. A. Teys, A. V. Dvurechenskii, O. M. Borodavchenko, V. D. Zhivulko, A. V. Mudryi

**Affiliations:** 1 Rzhanov Institute of Semiconductor Physics, SB RAS, 630090 Novosibirsk, Russia; 2 Novosibirsk State University, 630090 Novosibirsk, Russia; 3 Scientific-Practical Material Research Centre of the National Academy of Science of Belarus, P. Brovki, 220072 Minsk, Belarus

**Keywords:** Nanoscience and technology, Optics and photonics

## Abstract

The photoluminescence (PL) of the combined Ge/Si structures representing a combination of large (200–250 nm) GeSi disk-like quantum dots (nanodisks) and four-layered stacks of compact groups of smaller (30 nm) quantum dots grown in the strain field of nanodisks was studied. The multiple increase in the PL intensity was achieved by the variation of parameters of vertically aligned quantum dot groups. The experimental results were analyzed on the basis of calculations of energy spectra, electron and hole wave functions. It was found that the quantum dot arrangement in compact groups provides the effective electron localization in Δ_*x*,*y*_-valleys with an almost equal probability of finding an electron in the Si spacer and Ge barrier. As a result, the main channels of radiative recombination in the structures under study correspond to spatially direct optical transitions.

## Introduction

Currently, there is a considerable interest in the search for possible ways of creating the light-emitting devices based on the silicon technology^1–9^. Silicon is an indirect band gap material characterized by a low quantum efficiency, and one of the ways to overcome this problem is to use the structures with quantum dots (QDs), in which the strong carrier confinement and, consequently, the momentum uncertainty eliminates the prohibition of direct optical transitions. Ge/Si heterostructures with QDs are considered as one of the most promising systems for creating silicon-based light-emitting devices. There are a lot of works devoted to the study of the structures with GeSi QDs as the basis of light-emitting devices^6–11^. However, the problem remains unresolved. A significant downside of a GeSi QD system is the localization of holes and electrons at different sides from a heterointerface, i.e. belonging to type-II heterostructures, that results in small overlapping integrals and, consequently, in a small probability of radiative recombination. A noticeable increase in photoluminescence (PL) is obtained only in the structures with GeSi QDs embedded in microresonators^12–16^.

**Figure 1:**
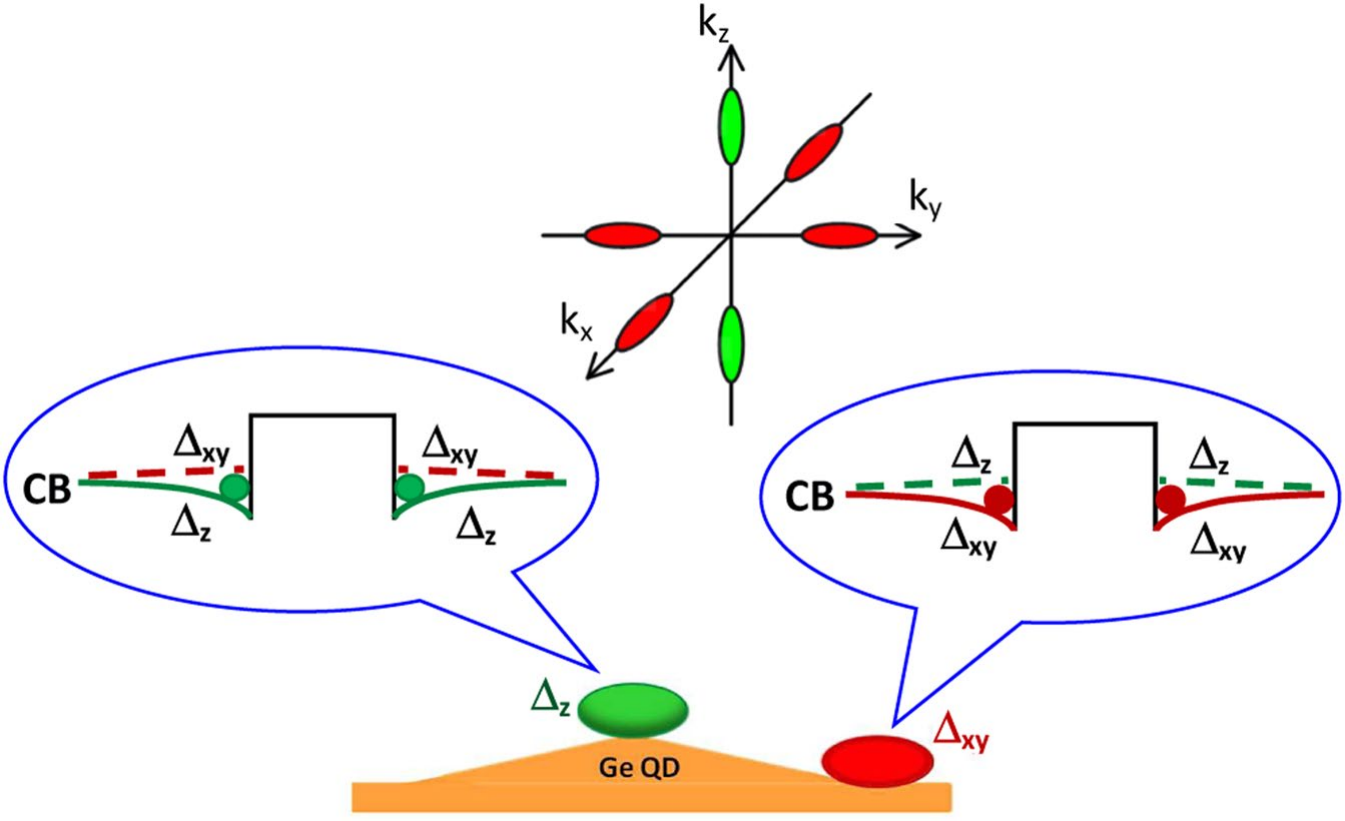
A scheme of the electron localization in the vicinity of Ge QD embedded in Si matrix. The green cloud at the apex of QD symbolizes a Δ_*z*_-valley electron, the red cloud at the QD base edge symbolizes a Δ_*x*,*y*_-valley electron. The upper part shows six Δ-valleys at the bottom of Si conduction band.

This paper is aimed at developing the light-emitting structures based on a Ge-Si heterosystem containing the compact groups of closely spaced QDs formed during the molecular beam epitaxy (MBE) on the strain-patterned substrates. The main idea is to use the built-in strain for controlling the spatial localization of charge carriers, their energy spectrum and overlapping between electron and hole wave functions. This is possible because of the exclusive role that the strain plays in the band alignment in Ge/Si heterostructures. Electrons can be localized in this system only due to the strain because the Ge-rich regions (QDs) represents potential barriers in the conduction band. Strain in surrounding Si causes a splitting of the six-fold degenerate conduction band edge at the Δ point and a separation of Δ_*z*_- and Δ_*x*,*y*_-valleys. Due to the inhomogeneous strain, the potential wells for electrons are formed in different Si regions near QDs, near the apexes of Ge QDs and at the periphery of QDs. The strain near QD apexes shifts Δ_*z*_-valleys down and promotes the localization of Δ_*z*_-valley electrons, while the opposite strain at the periphery of QDs leads to the localization of Δ_*x*,*y*_-valley electrons (Fig. 1). According to results obtained in ref. ^17^, the localization of Δ_*x*,*y*_-valley electrons is more preferential because it can provide a manyfold increase of PL intensity. The Δ_*x*,*y*_-valley electrons have a larger penetration into Ge regions and, correspondingly, a larger overlapping with holes localized in Ge. Such PL enhancement was revealed recently for double QD structures^17^, but only at the helium temperature due to a small binding energy of QD electron. In the present paper, the strain engineering allows us to develop the GeSi QD structures with larger binding energies of Δ_*x*,*y*_-valley electrons, providing the enhanced PL up to room temperature.

Recently it was shown that the effective localization of Δ_*x*,*y*_-valley electrons can be realized in compact QD groups with very close arrangement of QDs^18^. One of the ways to create these groups is the growth on the strain-patterned substrate with GeSi nanodisks embedded under the surface^19^. The strain field of the nanodisk plays a dual role: it creates nucleation sites for QD group, and contributes into balance between the band edges of different Δ-valleys. Changing the spacer thickness between the nanodisk layer and the growing QD group, one can control the spatial configuration of QD group and the potential well depth for electrons in different Δ-valleys^20,21^.

## Structures under study

The design of the QD structures is inspired by ideas of ordering self-assembled islands without substrate patterning, suggested by Capellini *et al*.^22^ and developed in refs. ^19,21,23^. The samples represent a combination of large (200–250 nm) GeSi disk-like quantum dots (nanodisks) and stacks of compact groups of smaller (30 nm) quantum dots grown in the strain field of nanodisks. The presence of large QDs provides a significant deformation in surrounding silicon, effectively lowering the conduction band edge and, thereby, forming a wide potential well. Small QDs, which have a higher Ge content, provide peak deformations, forming narrow and deep potential wells for electrons in silicon. However, if QD groups are grown at the surface of nanodisk, the effective localization of Δ_*x*,*y*_-valley electrons can not be realized because the strain field above the nanodisk promotes only the Δ_*z*_-valley electron localization. Therefore the QD group should be grown at some distance from the nanodisk^20^.

**Figure 2:**
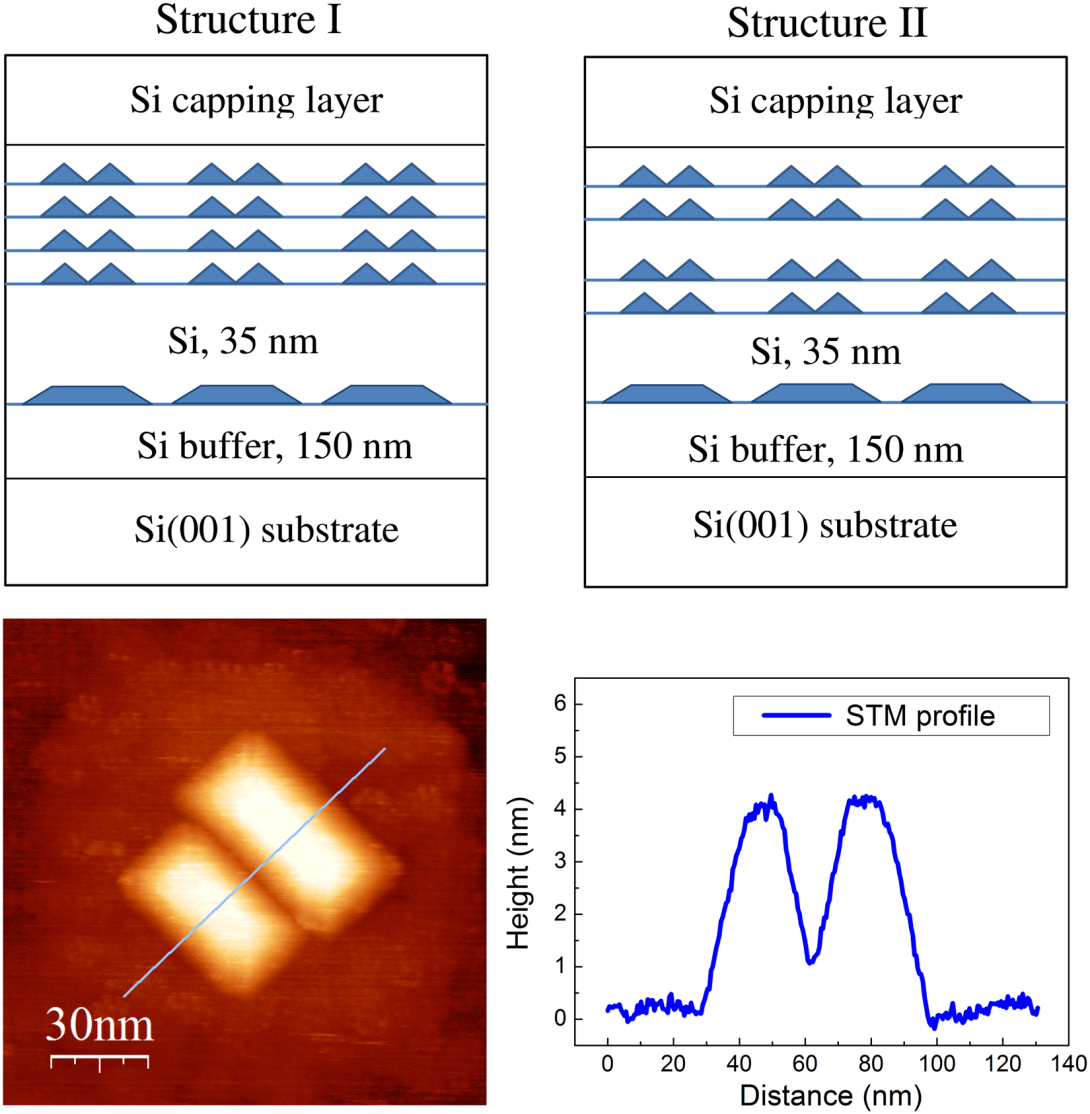
Top panels: a schematic structure of the samples under study. Bottom panels: STM image (150 ×150 nm^2^) and STM profile of QD pair in the fourth QD layer in stack grown at 580 °C on the substrate with GeSi nanodisks, incorporated under the surface at the depth of 35 nm and serving as templates for the nucleation of the QD groups. The image sides are oriented along $\langle 110\rangle$ directions.

The optimal structure represents a four-layered stack of compact QD groups incorporated in Si at the distance of 30–40 nm above the large GeSi nanodisks (structure I in Fig. 2). At this distance, the strain distribution on the surface of growing layer allows one to form the compact QD groups containing two or three coupled *hut*-clusters with the alignment of the longest QD edges. The thickness of spacer layers between QD layers was chosen to be equal to the QD height to obtain the greatest possible deformations in surrounding Si. Such QD groups configuration is very similar to the one observed in the double QD structures studied in ref. ^17^. However, it allows one to obtain QD electron states with one order larger binding energies and therefore to observe the PL signal up to room temperature.

In order to confirm that the origin of PL enhancement in the developed QD structure is the localization of electrons in Δ_*x*,*y*_-valleys, we grew the test structure with a varied thickness of spacer layer *d*: with *d* = 5 nm for the central spacer layer and *d* = 3 nm for the spacer layers between the first and second QD layers, and the third and fourth QD layers (structure II in Fig. 2). Such type of QD structure allows the localization of electrons not only in the Δ_*x*,*y*_-valleys, but in the Δ_*z*_-valley, that has been demonstrated recently in EPR experiments^21^. The fact that the part of electrons are relocated to the Δ_*z*_-valley, should lead to a decrease in the PL signal from QDs.

## Results and Discussion

**Figure 3:**
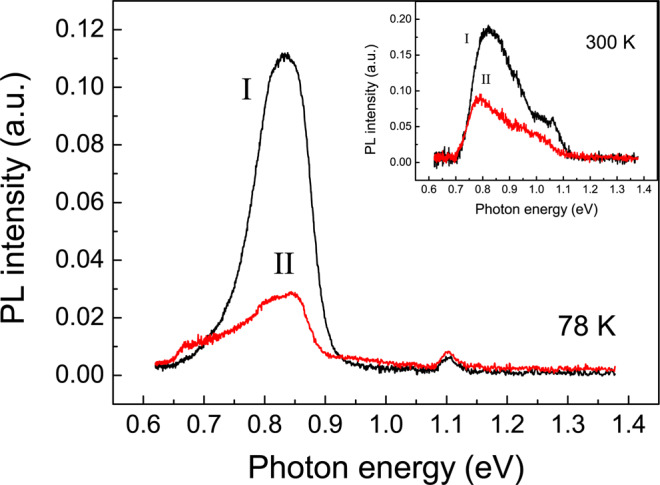
PL spectra of structure I (*d* = 3,3,3 nm) and structure II (*d* = 3,5,3 nm). The measurements were made at 78 K with a 532 nm excitation laser. The excitation power was 10 W/cm^2^. Inset: the PL spectra measured at 300 K with a 405 nm excitation laser. The PL spectra were recorded by using an InGaAs p-i-n photodiode (78 K) and a nitrogen cooled Ge detector (300 K).

The PL spectra of structures I and II measured at 78 K are shown in Fig. 3. Two PL peaks related to the radiative recombination in the QD groups (at 0.84 eV) and interband recombination in silicon (at 1.1 eV) were observed for both structures. The effect of PL enhancement, nearly four times (at 78 K), for structure I (*d* = 3,3,3 nm), as compared to structure II (*d* = 3,5,3 nm), was found. A less pronounced effect, a nearly twofold amplification, is observed at room temperature (see the inset in Fig. 3). To confirm the assumption that the effect observed is provided by different types of the electron spatial localization in the structures under study, we have calculated the eigenvalues and wave functions of electrons and holes. To perform the calculations, we consider the model of QD structure which is maximally close to a real one. For scanning tunneling microscopy (STM) studies, the test uncovered QD structures were grown in the same conditions. The STM study demonstrates that QDs have a *hut*-cluster shape with a height *h* ≈ 3 nm and lateral size *L*_*x*_ ≈ 30 nm (the width of *hut*-cluster base). The average length of long base edge is close to *L*_*y*_ ≈ 60 nm. QDs in groups have a very close arrangement. The STM profile of a typical QD pair is shown in Fig. 2 (right bottom panel). It is clearly seen that the neighboring QDs cross each other that can promote the hole tunneling between QDs and can lead to increasing the overlap integral between a hole and a Δ_*x*,*y*_-valley electron. We take for calculations the value of QD intersection of 5 nm.

The calculation results confirm that the value of overlap integral *I*_*eh*_ for the Δ_*x*,*y*_-valley electrons in structure I is larger than the one for the Δ_*z*_-valley electrons in structure II. The ratio of integrals is ≈ 1.4 (see details of calculations in Supplementary materials). The radiative recombination probability is proportional to ${I}_{eh}^{2}$. Hence one can expect a twofold exceeding of the PL intensity for structure I. To understand the obtained results, one needs to know the spatial location of the Δ_*x*,*y*_-valley electrons and the Δ_*z*_-valley electrons in the structures under study.

**Figure 4:**
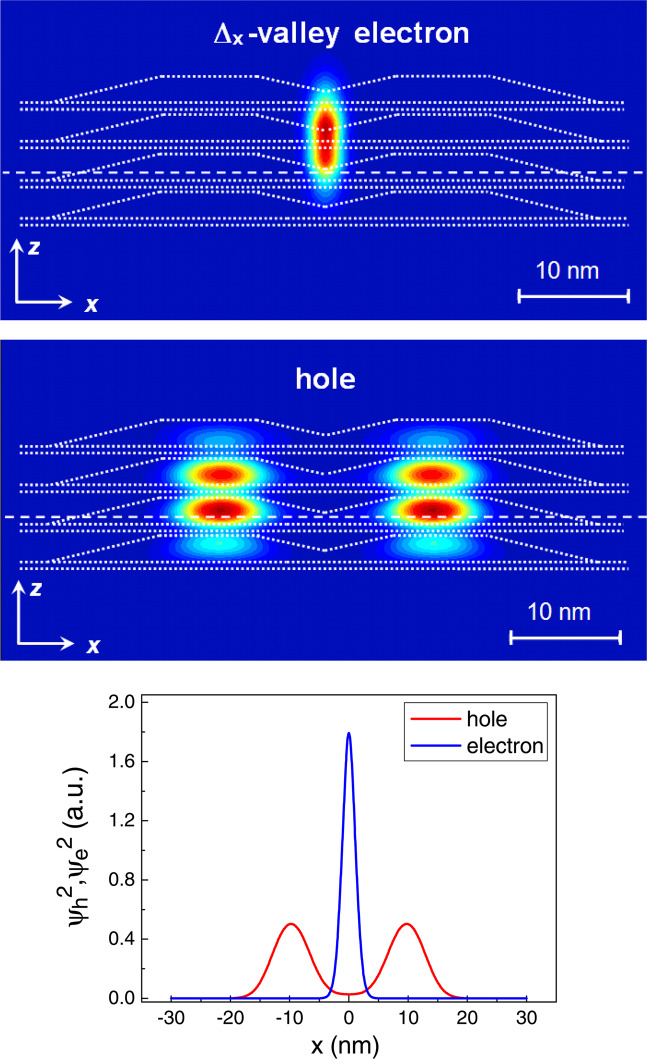
Wave functions of the Δ_*x*_-valley electron state (top panel) and hole ground state (center panel) in structure I (*d* = 3, 3, 3 nm). Here the Δ_*x*_-valley electron state localized at the QD base edge oriented along the *y*-axis is shown (correspondingly, the Δ_*y*_-valley electron is localized at QD base edge oriented along the *x*-axis). Sections of XZ plane passing through the center of the compact QD group are shown. The QD geometry is shown by dotted lines. A bottom panel shows the wave function profiles for an electron and a hole along the direction shown by the dashed line in the upper panels.

**Figure 5:**
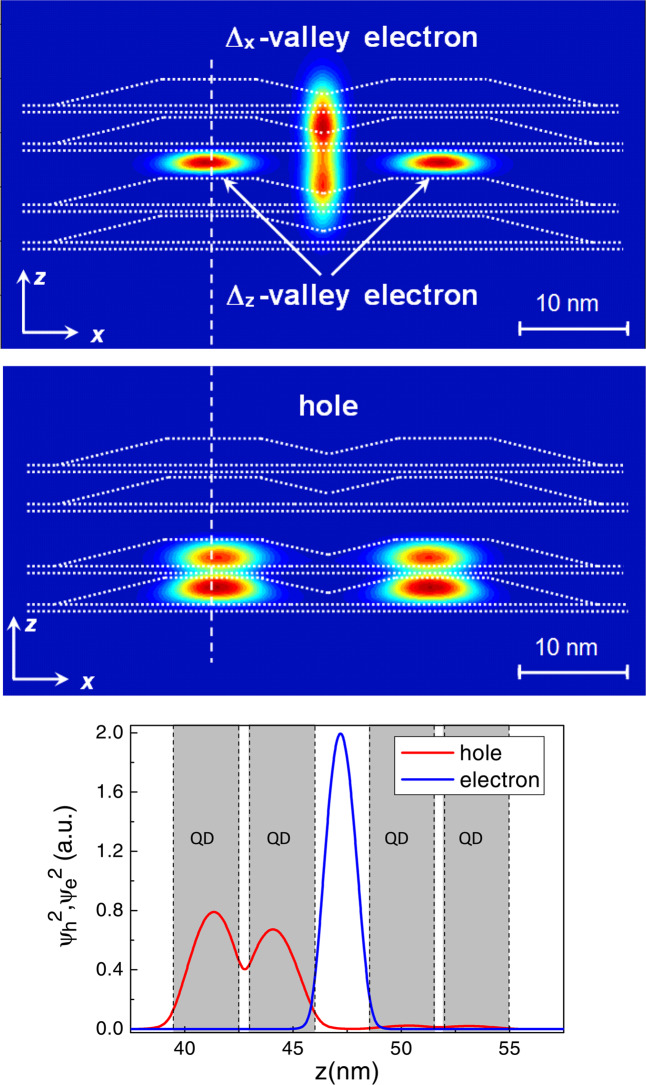
Wave functions of the Δ_*x*_- and Δ_*z*_-valley electron states (top panel) and the hole ground state (center panel) in structure II (*d* = 3, 5, 3 nm). Here, the Δ_*x*_-valley electron state localized at the QD base edge oriented along *y*-axis is shown (correspondingly, the Δ_*y*_-valley electron is localized at the QD base edge oriented along the *x*-axis). The sections of the XZ plane passing through the center of the compact QD group are shown. The QD geometry is shown by dotted lines. On the bottom panel are the wave function profiles for a Δ_*z*_-valley electron and a hole along the direction shown by the dashed line in the upper panels.

The Δ_*x*,*y*_-valley electron in both structures I and II is localized in the center of QD structure, just at the line of QD crossing (Figs. 4, 5). Such central position of electron provides the largest value of *I*_*eh*_ (in relative units ${I}_{eh}\approx 1.14\cdot {I}_{0}$) in structure I. The Δ_*x*,*y*_-valley electron in structure II has a smaller *I*_*eh*_ value (${I}_{eh}\approx 0.75\cdot {I}_{0}$), which is explained by the dislocation of the hole wave function in the stack. The hole wave function is mainly located in two lower QDs in the stack. This leads to increasing the distance between the centers of hole and electron wave functions, as compared to structure I and, consequently, to decreasing the *I*_*eh*_ value. The different distribution of hole in structures I and II is mainly due to different tunnel coupling between QD layers in stacks. The thin spacers in structure I provide the large tunneling integrals and this results in almost symmetric hole distribution along the QD stack. The increase in the central spacer thickness in structure II leads to the decrease of tunneling coupling between lower and higher pairs of QD layers. Therefore, a hole can be localized either in the lower pair or higher one. Due to the strain field of the nanodisk the hole ground state is located in two lower QD layers.

The Δ_*z*_-valley electron in structure II is localized in the central Si spacer near the QD apex (Fig. 5), very close to the hole ground state. However, it has a smaller *I*_*eh*_ value (${I}_{eh}\approx 0.81\cdot {I}_{0}$) than that of the Δ_*x*,*y*_-valley electron in structure I. This is a very interesting result because the distance *r*_*eh*_ between the centers of hole and electron wave functions differs approximately 5 times (for Δ_*x*,*y*_ electron ${r}_{eh}\approx 10$ nm, for Δ_*z*_ electron ${r}_{eh}\approx 2$ nm). Such a difference should ensure the exponential smallness of *I*_*eh*_ for the Δ_*x*,*y*_ electron, compared to the *I*_*eh*_ value for the Δ_*z*_ electron. But the numerical calculations demonstrate the opposite relation between overlap integrals. This result is a consequence of two factors. The first one is a higher value of the electron penetration into the Ge barrier $\eta \sim {\int }_{Ge}{\psi }_{e}^{2}dV$ for Δ_*x*,*y*_-valley electrons. For a finite potential well, $\eta \sim 1/{(\sqrt{{m}_{eff}})}^{3}$, where ${m}_{{\rm{eff}}}$ is the electron effective mass across the interface^17^. The effective mass difference for electrons in Δ_*z*_ and Δ_*x*,*y*_-valleys (in the first case ${m}_{{\rm{eff}}}={m}_{\parallel }$ and in the second case ${m}_{{\rm{eff}}}={m}_{\perp }$) provides one order larger penetration value for Δ_*x*,*y*_ electrons. The second factor is a QD crossing, that provides a sufficiently large probability of finding a hole in the center of QD structure (see Fig. 4). Indeed, the QD crossing results in a five orders larger hole probability density in the center of the structure (see comparison with the structure without QD crossing in Supplementary materials). As the result, the structure without QD crossing give a two orders smaller value of *I*_*eh*_ for the Δ_*x*,*y*_-valley electron (${I}_{eh}\approx 0.03\cdot {I}_{0}$). Thus, ${I}_{eh}^{2}$ is roughly proportional to the probability of finding a hole near an electron. So, the QD crossing is the main factor that determines the relation between *I*_*eh*_ values in our structures.

The electrons in Δ_*x*,*y*_- and Δ_*z*_-valleys can give a comparable contribution to the PL intensity in structure II because they have not only practically the same *I*_*eh*_ values, but very close binding energies (see Supplementary materials). However, the recent EPR study of analogous QD structures shows that the number of localized electrons in the Δ_*z*_-valley is 4–5 times smaller than the number of localized electrons in the Δ_*x*,*y*_-valley^21^. The authors supposed that the parameters of potential wells for electrons at the QD apexes in experimental QD structures differ from the expected ones. An insufficient depth or width results in decreasing the number of localized electrons. Since our structures were grown in the same growth conditions, we think that Δ_*z*_-valley electrons can give only small contribution to PL. To verify this assumption, we have studied the excitation power dependence of the PL spectra. This study can help to distinguish what kind of electrons determines the PL intensity. The optical transition with the participation of the Δ_*x*,*y*_ electrons can be considered as direct in space, because the electron wave functions are distributed over Si and Ge layers with an almost equal probability of finding an electron in the Si spacer and Ge barrier. In contrast, the recombination of Δ_*z*_ electrons with holes is a typical example of spatially indirect optical transitions, since the electrons and holes are localized at different sides of the Si/Ge interface. For spatially direct optical transitions the position of PL peak should not be changed with increasing the excitation power^24^. In the case of spatially indirect optical transitions, the blue shift of PL peak should be observed with increasing the excitation power^23,24^.

**Figure 6:**
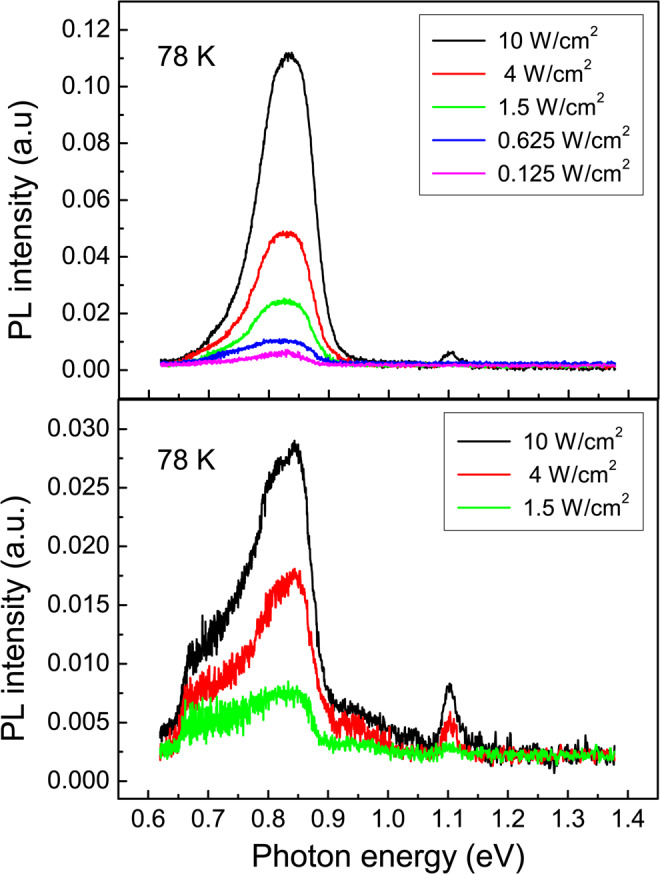
Excitation power dependent PL spectra of structure I (top panel) and structure II (bottom panel). The measurements were made at 78 K with a 532 nm excitation laser. The PL spectra were recorded using an InGaAs p-i-n photodiode.

The PL spectra dependence on the excitation power demonstrates that for both structures I and II the position of QD emission peak remains the same for all excitation power levels (Fig. 6).

Hence, the main recombination channel in both structures I and II should be a direct in space optical transition. Therefore we conclude that the recombination of holes with Δ_*x*,*y*_ electrons dominates in all structures under study. For structure II, the recombination of holes with Δ_*z*_ electrons gives only a small contribution to the PL spectra forming the low-energy shoulder of QD-related emission peak (Fig. 6, bottom panel).

Thus, the difference in PL intensities observed for structures I and II is defined by the difference in overlap integrals *I*_*eh*_ for the electrons in Δ_*x*,*y*_-valleys in these structures.

## Conclusions

In this work, we have developed the structures with stacks of compact GeSi QD groups, where PL is mainly provided by the recombination of holes with electrons in Δ_*x*,*y*_-valleys. Due to the special design of QD structures, the main optical transitions are spatially direct, which is confirmed by the PL spectra dependence on the excitation power. The PL intensity turns out to be very sensitive to the parameters of QD structures. A slight variation of the thickness of Si layers separating the QDs in the stacks results in the multiple increase of the PL intensity from QDs. The effect of PL enhancement is persistent up to room temperature due to the large strain in QD layers providing the effective electron localization. Based on the calculations of overlap integrals between the electron and hole wave functions, the difference in the PL intensities for the structures under study is explained. It was found that the QD crossing is crucial for obtaining the PL amplification effect. The structures with a QD crossing provide a sufficiently large probability of finding a hole in the center of QD structure and, correspondingly, a significant increase in overlap integrals.

## Methods

We grew the experimental structures by MBE on n-Si(001) substrates with a resistivity ≥ 1000 Ω cm. Firstly, a 100 nm buffer Si layer was grown at *T* = 500 °C. The nanodisk layer was grown by the deposition of 7.5 Ge ML at 700 °C. Then a 35 nm Si spacer layer was grown at 700 °C. Each QD group layer was grown at the temperature ${T}_{QD}$= 580 °C. The first QD layer was formed by the deposition of 5.5 Ge ML. The Ge QDs formation in each subsequent layer was controlled by the reflection high energy electron diffraction (RHEED). A moment, when the RHEED pattern changes to spotty, is considered as beginning of three-dimensional island formation, after which a 0.3 Ge ML was deposited additionally. Such procedure provides an almost equal size of Ge QDs in all layers of the stacked structure^25,26^. Si spacer layers were grown at 400 °C. The top QD layer was covered by the 5 nm Si layer also at 400 °C to preserve the QD shape. Finally, all structures were covered at 500 °C by the 195 nm Si layer doped by Sb with a concentration $\simeq 5\cdot {10}^{16}$ cm^−3^ to supply the QD layers by electrons. Such thickness and doping level of the Si top layer allow filling QDs by electrons and checking the electron spatial localization by EPR measurements, like in ref. ^21^.

The eigenvalue problem was solved using the nextnano^3^ software (ref. ^27^). This program allows one to take the strain effects and the real geometry of nano-objects into account. The calculation of the strain distribution was performed using the analytical expressions developed in the ref. ^28^ and program Easystrain3d (see ref. ^29^), that reduced significantly (by orders of magnitude) the calculation time. All electron energies were obtained in the one-electron approximation using the effective mass method. The hole energies were determined using the $6\times 6\,{\rm{kp}}$ method. The QD parameters for calculations were chosen based on the data obtained by atomic force microscopy (AFM), STM, transmission electron microscopy (TEM) and extended X-ray absorption fine structure (EXAFS) spectroscopy measurements^30^.

## Supplementary information

Supplementary information.
