# Supplementary material for: Photoluminescence of compact GeSi quantum dot groups with increased probability of finding an electron in Ge
Source: Sci Rep. 2020 Jun 9;10:9308. doi: 10.1038/s41598-020-64098-x (PMC7283240; doi:10.1038/s41598-020-64098-x)
Supplement: Supplementary file 1 — Supplementary information. [file 41598_2020_64098_MOESM1_ESM.pdf]

Supplementary materials to paper "Photoluminescence of compact GeSi quantum dot groups with increased probability of finding an electron in Ge"

A. F. Zinovieva[a,b,*] V. A. Zinovyev[a], A. V. Nenashev[a,b], S. A. Teys[a], A. V. Dvurechenskii[a,b], O. M. Borodavchenko[c], V. D. Zhivulko[c], and A. V. Mudryi[c]
[a]*Rzhanov Institute of Semiconductor Physics, SB RAS, 630090 Novosibirsk, Russia*
[b]*Novosibirsk State University, 630090 Novosibirsk, Russia*
[c]*Scientific-Practical Material Research Centre of the National Academy of Science of Belarus, P. Brovki 19, 220072 Minsk, Belarus*
(Dated: December 28, 2019)

The eigenvalue problem was solved using the nextnano[3] software (Ref. 1). This program allows one to take into account the strain effects and the real geometry of nano-objects. The calculation of the strain distribution was performed using analytical expressions developed in the work[2] and program Easystrain3d (see Ref. 3), that reduced significantly (by orders of magnitude) the calculation time. The QD parameters for calculations were chosen based on the data obtained by atomic force microscopy (AFM), STM, TEM, and extended X-ray absorption fine structure (EXAFS) spectroscopy measurements. Strain of large disk-like QDs was taken into account. The shape of nanodisks was described by the truncated cone with the bottom diameter 250 nm, top diameter 200 nm and height 10 nm. Ge content x was taken for nanodisks to be equal 0.35, while for *hut*-clusters $x = 0.5$. To perform the calculations, we consider the model of QD structure which is maximally close to a real one. For scanning tunneling microscopy (STM) studies, the test uncovered QD structures were grown in the same conditions. It was found that QDs have a height $h \approx 3$ nm and lateral size $L_x \approx 30$ nm (the width of *hut*-cluster base). The average length of long base edge is close to $L_y \approx 60$ nm. We took into account the effect of QD apex smearing at the overgrowth of QDs. Then the height of QDs was decreased down to $h = 2.5$ nm, i.e. *hut*-clusters were truncated (see the results of transmission electron microscopy (TEM) study of the similar structures in Ref. 4). The inclination angle of side facets corresponds to facet orientation {105}. Also, it was established that QDs in groups have a very close arrangement. The STM profile of a typical QD pair is shown in Fig. 1 of the main paper (right bottom panel). It is clearly seen that the neighboring QDs cross each other that can promote the hole tunneling between QDs. On the average, the thickness of wetting layer (WL) in the contact point increases up to $h_{WL} \approx 1.5$ nm, instead of the usual WL thickness $h_{WL} \approx 0.5$ nm. We model two cases: (1) QD pairs with a crossing, the length of QD intersection $\Delta L = 5$ nm, $h_{WL} = 1.5$ nm, (2) QD pairs without crossing, $h_{WL} = 0.5$ nm. Two model structures corresponding to experimental structures I and II were considered. The similar calculations were performed in the work,[5] but without WL and QD crossing. For electron states WL does not strongly affect the electronic structure, but for holes the presence of WL is very important.

All electron energies were obtained in one-electron approximation using effective mass method. Hole energies were determined using the $6 \times 6\,\mathbf{kp}$ method. Integrals of overlapping between electron and hole wave functions were estimated as follows:

$$I_{eh} = \int \sqrt{\rho_e(\mathbf{r})}\sqrt{\rho_h(\mathbf{r})}\mathrm{d}V, \tag{1}$$

where $\rho_e(\mathbf{r})$ and $\rho_h(\mathbf{r})$ are electron and hole wave function density distributions normalized as $\int \rho_e(\mathbf{r})\mathrm{d}V = \int \rho_h(\mathbf{r})\mathrm{d}V = 1$. This estimation takes into account the spatial separation of electrons and holes, but does not account for their separation in k-space natural for indirect band-gap materials, Si and Ge. Therefore, only the ratios of these integrals are physically meaningful.

To calculate the energy of the electron in the presence of the hole localized in QD stack, we add to the Hamiltonian the term $U_c = e^2/(4\pi\varepsilon\varepsilon_0\sqrt{(x-x_0)^2+(y-y_0)^2+(z-z_0)^2+a^2})$, where $\varepsilon = 12$ is the relative permittivity of Si, (x_0, y_0, z_0) is the position of the hole wave function maximum, a is the smallest size of this wave function (along z-direction), $a = a_z \approx 2$ nm. To take into account the hole distribution between QDs we put one half of the hole charge in each QD in pair.

The results of calculations are summarized in Table I, where the calculated values of overlap integrals and binding energies for $\Delta_{x,y}$-valley and Δ_z-valley electrons are given. In contrast to the simple case of hole localization inside GeSi QDs (in Γ point), the inhomogeneous strain distribution in our structures results in the formation of many potential wells in different Δ-valleys. For clarity, we draw the scheme of electron potential well arrangement for structure II (see Fig. 1).

For structure I ($d = 3, 3, 3$ nm) the ground electron state in the $\Delta_{x,y}$-valley is localized near the base edges of QDs. In the case of the structure without QD crossing, the ground electron state is found in the center of QD structure (site **D** in Fig. 1) in a good correspondence to the results of work.[5] The binding energy E_b of such electrons is approximately 66 meV. The QD crossing changes the strain distribution, decreasing the strain near the point of QD crossing. Then the potential well depth

TABLE I: Binding energies E_b and integrals of overlapping with ground hole state I_{eh} for the electrons in different Δ-valleys localized in the structures with a QD crossing ($\Delta L = 5$ nm). $E_b^{(0)}$ (E_b) are calculated without hole (with hole). Site **A** is absent in the case of structure I, because the corresponding Si spacer is too thin. The energies differ from values of E_b in Ref. 5, where the structures without QD crossing (moreover, with the gap $b = 3$ nm between QD base edges) were considered.

structure	valley	site	$E_b^{(0)}$ (meV)	E_b (meV)	$I_{eh}(\times 10^{-1})$
I d=3,3,3 nm	Δ_z	A	–	–	–
		B	44	49	0.271
		C	36	44	0.274
	$\Delta_{x,y}$	D	**30**	**40**	**1.14**
		E	33	38	0.018
II d=3,5,3 nm	Δ_z	A	42	49	0.81
		B	39	45	0.09
		C	33	44	0.51
	$\Delta_{x,y}$	D	**21**	**30**	**0.75**
		E	29	33	0.014

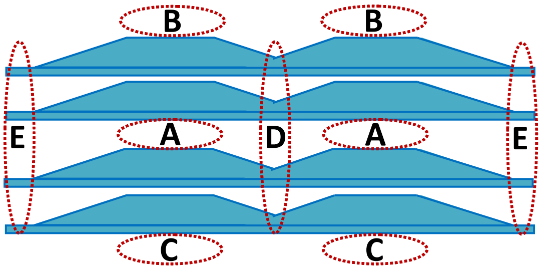

FIG. 1: Scheme of electron potential well arrangement in the structures under study. **A**, **B**, **C** potential wells are formed in the Δ_z valley. **D**, **E** localization sites correspond to electrons in the $\Delta_{x,y}$ valley. Site **A** is absent in structure I.

and E_b for the electron localized at site **D** are reduced. In the structure with $\Delta L = 5$ nm the binding energy is decreased down to ≈ 30 meV (see Table I). However, if one takes into account the presence of the hole, localized in the QD stack (see the hole ground state in Fig. 4 of the main paper) and the Coulomb interaction with this hole, then the electron binding energy is restored up to a value ≈ 40 meV. The $\Delta_{x,y}$-electron wave function is distributed over Si and Ge layers with practically equal probability of finding an electron in the Si spacer and Ge barrier, i.e. the recombination of this electron with a hole can be considered as a direct in space transition.

The electrons at the external edges of QDs (site **E**) have slightly larger binding energies (for example, $E_b \approx 33$ meV in the structure with QD crossing), but two orders smaller overlap integrals with the hole ground state. Then they cannot give a sufficient contribution to PL. The ground electron state in the Δ_z-valley is localized near the apex of QDs at the top of QD group (site **B**). The similar electron state is localized under the bottom of QD group (site **C**). The binding energy of these electrons are 44 meV and 36 meV (in the absence of hole), corre-

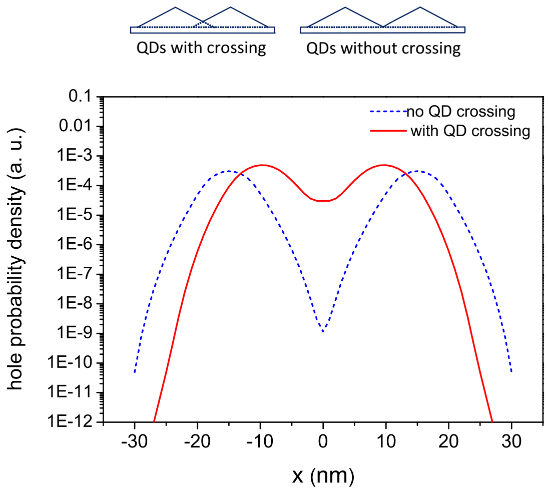

FIG. 2: X-profiles of squared hole wave function for structure I with (without) QD crossing. Profiles are taken along the line connecting the left and right QDs in the third QD layer (from bottom of QD structure).

spondingly. Despite the Δ_z-valley electrons are found closer to the hole state, the overlap integral is several times less than the one for the $\Delta_{x,y}$-electron (see Table I). This result is provided by a redistribution of the ground hole state between QDs in the x-direction (see Fig. 3 in the main paper).

In the case of structure II ($d = 3, 5, 3$ nm), the ground electron state in the Δ_z valley is localized near the apex of QD in the central Si spacer with d=5 nm (site **A**). The electron binding energy, in this case, is 47 meV (for the structure without QD crossing). For the structure with $\Delta L = 5$ nm, this energy is decreased down to 42 meV. The presence of the hole leads to increasing the value of E_b up to 49 meV. The central position of this electron state should provide the large overlap integral, but the hole wave function is located practically in two lower QDs, and it sufficiently decreases I_{eh}.

As for the $\Delta_{x,y}$-electron localized in the center of structure II, its binding energy is smaller than in the case of structure I. Without QD crossing $E_b \approx 55$ meV, while in the structure with a QD crossing $E_b \approx 21$ meV. The presence of hole increases the value of E_b approximately by 9 meV, resulting in $E_b = 30$ meV. The I_{eh} value is comparable with the one for the Δ_z-electron (at site **A**). The electron wave function is also distributed over Si and Ge layers with practically equal probability of finding an electron in the Si spacer and Ge barrier (see Fig. 4 in the main paper), like in structure I, then the optical transition with the participation of this electron can also be considered as direct in space.

Results of calculation demonstrate that the overlap integral between the hole ground state and $\Delta_{x,y}$ electron state is very sensitive to the presence of QD crossing. The I_{eh} value for the structure without QD crossing (but with WL) is two orders smaller than that for the structure with QD crossing. The probability of finding a hole

in the center of QD structure strongly increases in the case of QD crossing (Fig. 2).

* aigul@isp.nsc.ru

[1] See http://www.nextnano.de/nextnano3/ for information about the program nextnano^3.
[2] A. V. Nenashev and A. V. Dvurechenskii, J. Appl. Phys. **107**, 064322 (2010).
[3] See http://easystrain.narod.ru/ for information about strain calculation using analytical approach.
[4] V. A. Zinovyev, A. F. Zinovieva, P. A. Kuchinskaya, Zh. V. Smagina, V. A. Armbrister, A. V. Dvurechenskii, O. M. Borodavchenko, V. D. Zhivulko, and A. V. Mudryi, Appl. Phys. Lett. **110**, 102101 (2017).
[5] A. F. Zinovieva, V. A. Zinovyev, A. V. Nenashev, L. V. Kulik, A. V. Dvurechenskii, Phys. Rev. B **99**, 115314 (2019).
